# Improving Tribological Properties of 45-Steel by Combining Laser Surface Texturing and DLC Coating

**DOI:** 10.3390/ma19194115

**Published:** 2026-09-26

**Authors:** Yixu Niu, Xianjuan Pang, Ruofan Zhao

**Affiliations:** 1 School of Intelligent Manufacturing, Luoyang Institute of Science and Technology, Luoyang 471023, China; niuniuyixu@163.com; 2 National United Engineering Laboratory for Advanced Bearing Tribology, Henan University of Science and Technology, Luoyang 471023, China; rf2022hhxx@163.com; 3 School of Materials Science and Engineering, Henan University of Science and Technology, Luoyang 471023, China

**Keywords:** tribological properties, laser surface texturing, diamond-like carbon coating

## Abstract

In efforts to improve the tribological properties of the 45-steel, laser surface texturing (LST) and diamond-like carbon (DLC) coating were performed on the surfaces of the steel. Three types of samples, i.e., LST followed by DLC coating (LST-C), DLC coating followed by LST (C-LST) and DLC coating without LST(C-only) were prepared. The tribological properties of the samples were investigated under dry friction and starved lubrication, respectively. The results show that compared to coating-only sample, the duplex-treatment can significantly reduce the friction coefficient and wear rate, among which LST-C samples demonstrate the best tribological performance both under dry friction and starved lubrication. DLC coating can prolong the existence time of the surface textures. Meanwhile, the dimple-textures can capture the wear debris and store lubricating oil, which help to reduce the abrasive wear and extend the existence time of stable state.

## 1. Introduction

The 45-steel, as a medium carbon steel, is widely used in important structural parts because of its excellent mechanical properties. However, low surface hardness and relatively poor wear resistance limit the application of 45-steel. Among various surface treatment methods, laser surface texturing (LST) is one of the most commonly used surface modification methods, which refers to the use of laser ablation to process a non-smooth surface with a certain morphology on the material surfaces [1,2,3]. Laser surface texturing typically has the advantages of high precision, fast processing speed, no pollution, silent processing process, and no deformation of the workpiece. The processed dimples and grooves can collect grinding debris, thereby reducing abrasive wear [4,5]. Under oil lubrication conditions, texturing can store lubricating oil, increase oil film thickness, generate fluid dynamic pressure to improve oil film bearing capacity [6], and extend oil film existence time [7,8].

In addition to laser surface texture, surface coating technology is another commonly used surface modification technology, which refers to the use of certain coating equipment and processes to generate coating materials on the substrate material, with the aim of improving the surface properties of the substrate material and increasing mechanical strength. As is well known that the diamond-like carbon (DLC) coating has an amorphous three-dimensional network structure that mixes Sp3 hybridized carbon bonds with Sp2 hybridized carbon bonds [9,10], which shows excellent tribological properties such as high hardness, low coefficient of friction, and good chemical inertness [11]. It is widely used in mechanical, optical, electronic, biomedical and other fields [12,13]. At present, the research on DLC coatings mainly focuses on the aspects of composition [14,15], structure [16,17], preparation methods [18,19], properties [20,21,22], and applications [23,24]. 

The two surface modification techniques can interact with each other [25], and the ablation of the laser can affect the microstructure and composition of the film, which may lead to partial graphitization of the DLC film [26], thereby affecting the film’s performance. According to our previous research, the optimized LST has a positive effect on improving the tribological properties of 45-steel surfaces, but it is difficult to further improve the performance by solely relying on the single treatment method [27]. Shi et al. [28] investigated the synergistic effects of laser-textured microgroove geometry and DLC coating on SUS304 stainless steel. The results showed that the 45° inclined groove structure exhibited stable friction coefficients within the 2–8 N load range, with finite element simulation revealing that residual stress in the DLC-TI sample was significantly lower than in other micro-texture types. Zhang et al. [29] studied bio-inspired micro-textures (hexagonal, rhombic, sectorial, and annular sector) combined with DLC coatings on SUS304 stainless steel. It was found that the hexagonal micro-texture/DLC coating surface exhibited the lowest and most stable coefficient of friction (0.17) along with minimal wear. A duplex-treatment may be a feasible way to significantly improve the tribological performance of 45-steel surfaces by combining laser surface texturing and DLC coating. Bai et al. [30], who reported that the textured surface with DLC coating reduced the stable friction coefficient by 59% compared to smooth surfaces under lubricated conditions. While the combination of LST and DLC coating has been studied for various materials, the specific influence of processing sequence—whether LST is performed before or after DLC deposition—on the tribological properties of 45-steel has not been systematically investigated. 

Three types of samples were prepared in this paper. One was treated by DLC coating first, and then micro-dimples were processed on the coatings by LST. Conversely, another one was treated by LST first, and followed by DLC coating. The third was only treated by DLC coating as the control group. The tribological tests were carried out under dry friction and starved lubrication respectively aiming to study the wear-reducing mechanism of DLC coating and LST treatment.

## 2. Materials and Methods

### 2.1. Laser Surface Texturing and DLC Coating 

The substrate material was 45-steel with a diameter of 28 mm and thickness of 10 mm. The major chemical composition of the steel is shown in Table 1.

Before the test, the surfaces of the steel were ground using sandpapers ranging from 240# to 1500#, and then polished using a MP-2B type metallographic polishing machine to obtain a surface roughness of Ra ≈ 0.08–0.12 μm prior to DLC coating. The samples were ultrasonically cleaned in anhydrous ethanol for 10 min.

The square arrays of micro-dimples with a texture density of 10%, a depth of 10 μm and a diameter of 100 μm were induced on the samples by using a FPS31-type nanosecond laser equipment. The laser processing parameters are as shown in Table 2. Each dimple was produced by applying 10 laser pulses at a fixed dwell time. The laser beam was focused through an F-theta lens (focal length = 100 mm) onto the sample surface at normal incidence.

After laser surface texturing, a gentle polishing process was used to remove bulges around the rim of the dimples. The profile of the dimpled-textured surfaces was characterized by a three-dimension profilometer (Nano Focus μsurf-expert, Nano Focus AG, Oberhausen, Germany).

DLC coating was deposited on the samples by the close-field magnetron sputtering technique. All samples were ultrasonically degreased with acetone for 10 min and cleaned with Ar^+^ ions at a substrate pulsed bias of −500 V for 10 min before deposition. Subsequently, a thin tungsten interlayer was deposited on the steel to improve adhesion with a W target at a power of 200 W for 10 min under an Ar atmosphere (estimated thickness ~120 nm). The DLC coating was then deposited at a substrate pulsed bias of −60 V, a frequency of 200 kHz, and a process pressure of 0.4 Pa under Ar flow (30 sccm) with high-purity graphite targets. The deposition time was 60 min, resulting in a coating thickness of approximately 1.4 μm. The substrate temperature was maintained at room temperature (~25 °C) without intentional heating. The working gas was high-purity argon (99.999%), and no reactive gases were used; therefore, the deposited coating is a non-hydrogenated DLC film. The surface roughness (Ra) of the DLC-coated sample was measured to be 0.10–0.15 μm. 

By using the above methods, three types of samples, i.e., LST followed by DLC coating (LST-C), DLC coating followed by LST (C-LST) and DLC coating without LST (C-only) were prepared for tribological tests. The processing sequence for each specimen is described in detail in Table 3.

### 2.2. Tribological Tests

Tribological tests were carried out on the MFT-5000 multi-function friction and wear testing machine (Rtec Instruments, San Jose, CA, USA), as shown in Figure 1, using ball-disk contact under dry friction and starved lubrication, respectively. The upper hard alloy(Chinese trademark is YG6) ball with a diameter of 6.35 mm fixed by the upper jig was kept stationary and in contact against a rotating lower sample, i.e., the prepared 45-steel samples. 

The major chemical composition of the YG6 hard alloy ball is 94wt.% W and 6wt.% Co. The applied normal load was 70N with a rotation speed of 500 rpm. The rotation radius was 4, 6 and 8 mm, so the corresponding sliding speed was 0.21, 0.31 and 0.42 m/s. All the samples were tested until the friction coefficient occurred a sharp increase at which the friction coefficient exceeds 0.3. Because the failure time differed among the three sample types and among the different sliding speeds, the test duration varied accordingly.

Starved lubrication refers to a lubrication regime in which the amount of lubricant supplied to the contact interface is insufficient to maintain a continuous, fully flooded oil film throughout the test. In this study, starved lubrication conditions were created by applying 4 μL of PAO4 oil to the contact point between the ball and the disc using a micropipette before the test. The physical properties of the oil are shown in Table 4.

The oil was evenly spread using a rubber applicator. No further lubricant was added during the test. As the test proceeded, the lubricant was gradually consumed through displacement, evaporation, and degradation, leading to a progressive transition from partial lubrication to boundary lubrication and eventually to severe wear. The test was continued until the friction coefficient(abbreviated as COF) exhibited a sharp increase (defined as COF > 0.3), which marks the failure of the protective surface. Each test was repeated three times under identical conditions. The wear rate of the discs was calculated according to the following formula:

w = V/(P∙L)
(1)

where V is the wear volume, mm^3^; P is the normal load, N; L is the sliding distance, m). The wear volume (V) of the disc samples was measured using a 3D profilometer (Nano Focus μsurf-expert, Nano Focus AG, Oberhausen, Germany) equipped with the manufacturer’s proprietary analysis software (μsoft analysis module, Version 3.2). For each worn track, five cross-sectional profiles were acquired at equally spaced positions along the wear scar. The wear cross-sectional area was determined by integrating the depth profile relative to the unworn surface baseline. The wear volume was then calculated by multiplying the average cross-sectional area by the total length of the wear track circumference. To ensure statistical reliability, each wear volume measurement was repeated three times. The typical relative standard error for the wear volume measurements was within ± 5–8%, depending on the wear severity.

### 2.3. Mechanical and Microstructural Characterization 

The hardness of the as-prepared surfaces was observed by an HV-1000 Vickers hardness tester (Huaying test instrument Co., Ltd., Shanghai, China) under a load of 0.2 kg and duration of 15 s. Each sample was measured at least five times. After the tribological tests, the worn surfaces were characterized by the three-dimension profilometer, and the micromorphology was analyzed by the SEM and Energy Dispersive Spectrometer (EDS, EDAX Octane SDD Series, EDAX Inc., Mahwah, NJ, USA). The structural properties of the DLC films were measured by micro-Raman spectroscopy equipped with a 532 nm argon ion laser from 600 to 2000 cm^−1^. And also, the temperature around the contact area was measured by using a FLIR A615 infrared thermal camera (FLIR Systems, Inc., Wilsonville, OR, USA).

## 3. Results

### 3.1. Profile of the Textured Surface and Characterization of DLC Coating

The hardness of the DLC-coated samples can get to 2210 HV_0.2_, while the hardness of the 45-steel substrate is about 201 HV_0.2_. It can be seen that DLC processing has a great impact on the hardness of the samples. After DLC treatment, the hardness of the substrate material increased by more than ten times.

The three-dimensional morphology and profiles of the textured samples are shown in Figure 2. It can be seen in Figure 2a that the dimpled textures are uniformly distributed on the surface in the form of a square array. Figure 2b,c show the profile of the C-LST and LST-C samples. Obviously, the diameter of the dimples is 100 ± 5 μm and the depth is 10 ± 2 μm. The actual sizes of the dimples are consistent with the set values, which indicates the high accuracy of laser processing. 

The cross-sectional SEM image of the DLC coating (C-only sample) is shown in Figure 3a. It can be seen that the DLC coating is composed of parallel columnar structures, which are perpendicular to the substrate and have a thickness of approximately 1.4 μm. Figure 2b shows the surface morphology of the coating surface, indicating that the film surface is relatively smooth, with some small defects distributed on it. The Raman spectrum result is shown in Figure 2c. It is clear that the D peak and G peak positions are located at approximately 1360 cm^−1^ and 1540 cm^−1^, with an ID/IG ratio of 0.88, which is typical of the Raman spectrum double peak position of DLC films.

The element distribution of the C-LST sample is shown in Figure 4. Fe and O elements are enriched at the center of the texture, which is the exposing the 45-steel substrate and accompanied by slight oxidation due to laser ablation. C and W elements are relatively less distributed in the texture pits, but evenly distributed on the unablated surface. It seems that the surface textures have disrupted the continuity of the DLC film.

### 3.2. Tribological Properties

#### 3.2.1. Dry Friction

The friction coefficient of the samples in different sliding speeds under dry friction is shown in Figure 5. The change in friction coefficient of all the samples follows a similar trend: initially maintaining a low and stable state (COF ≈ 0.10–0.20), then gradually increasing, and finally undergoing a sharp increase (COF > 0.3). The C-only sample has the shortest stable state before failure, while both C-LST and LST-C samples maintain a longer stable state than the C-only sample at all three sliding speeds. It can be seen that the change in friction coefficient of all the samples follows a similar trend, initially maintaining a low and stable state, then gradually increasing, and finally suddenly rising sharply. The C-only sample has the shortest stable state before failure, while both C-LST and LST-C samples keep a longer stable state than that of the C-only sample in the three sliding speeds. In addition, the LST-C sample has the longest low-friction state time, which is about six times that of the C-only sample which has a sliding speed of 0.31 m/s. 

Figure 6 shows the wear rate of the samples in different sliding speeds under dry friction. Each data point represents the mean of three independent tests, and the error bars represent the standard deviation. The C-only sample has the highest wear rate among the three types of samples in different sliding speeds. Compared with the C-only sample, the wear rate of the C-LST sample decreases by 87.9%, and that of the LST-C sample decreases by 96.4% for a sliding speed of 0.21 m/s. Moreover, the wear rate of the C-LST sample decreases by 82.8%, and the wear rate of the LST-C sample decreases by 84.6% compared with the C-only sample for a sliding speed of 0.31 m/s. Although the wear rate of the C-only sample significantly decreases for a sliding speed of 0.42 m/s, the wear rate of the C-LST and LST-C samples is still lower than it, which decreased by 21.4% and 33.5%, respectively.

#### 3.2.2. Starved Lubrication

The friction coefficient of the samples under starved lubrication in different sliding speeds is shown in Figure 7. As can be seen, the change in friction coefficient follows the same trend as dry friction, initially maintaining a low-stable state, then gradually increasing, and finally suddenly rising sharply. In our previous study, although the samples that were only treated by LST showed a lower fiction coefficient than the 45-steel substrate, the duration of the low-friction state only lasted for 600 s [27]. However, the C-only sample can keep the stable state for at least 20,000 s, which is the shortest time in comparison with C-LST and LST-C samples in this research. In the three sliding speeds, the LST-C sample always keeps the longest low-friction state time, which is about 1.25 times of the C-only sample for a sliding speed of 0.31 m/s.

Figure 8 shows the wear rate of the samples with different sliding speeds under starved lubrication. The change trend of wear rate is consistent with that of dry friction. The wear rate of C-only samples decreases with the increase in sliding speed. Compared to C-only samples, the wear rate of C-LST samples decreases by 92.9%, and LST-C samples decreases by 94.5% with a sliding speed of 0.21 m/s. For a sliding speed of 0.31 m/s, the wear rate of C-LST and LST-C samples decreases by 76.4%and 79.7%, respectively, compared with C-only samples. In addition, for a sliding speed of 0.42 m/s, the wear rate of C-LST and LST-C samples decreases by 53.1% and 55.8%, respectively, compared with C-only samples. Moreover, it can be seen that, compared with the dry friction, the friction coefficient of the samples under starved lubrication does not significantly decrease, but the wear rate decreases. This indicates that PAO4 lubricating oil has limited effect on reducing friction, but it plays a good role in improving wear resistance.

Figure 9 shows the microscopic morphologies of the C-only and LST-C samples at different sliding times under starved lubrication. For the C-only sample (Figure 9(a1–a3)), the DLC film undergoes progressive deformation and fracture as sliding time increases. In the initial wear stage (Figure 9(a1)), large flaky delamination pits appear, and parts of the DLC coating are broken by abrasive particles, though the coating still provides partial protection. In the middle stage (Figure 9(a2)), the coating is further fragmented into smaller pieces, and its continuity is severely disrupted, with debris accumulating on the worn surface. Finally (Figure 9(a3)), the coating is severely deformed and completely broken down, resulting in a rough, granular surface dominated by substrate material and oxidized wear products, and the coating loses its wear resistance.

In contrast, the LST-C sample (Figure 9(b1–b3)) exhibits a clear difference in wear evolution due to the presence of laser-textured dimples. In the initial stage (Figure 9(b1)), the dimple remains well-defined and acts as a trap for wear debris, while the surrounding DLC coating stays largely intact. In the middle stage (Figure 9(b2)), the dimple becomes heavily filled with compacted debris, and although some cracking and local spalling occur around the texture, the dimple structure is preserved and continues to reduce abrasive wear. In the final stage (Figure 9(b3)), the dimple is almost completely filled and covered by debris, and partial coating delamination is observed; however, the extent of coating failure is significantly less severe than that of the C-only sample. These observations confirm that the laser textures effectively delay coating breakdown and extend the wear life of the DLC coating under starved lubrication.

The temperature around the contract area was measured during the friction process. Figure 10a,b show the infrared thermal images of the LST-C sample before and after the friction coefficient rises sharply. Before the sharp increase (Figure 10a), the temperature around the contact area is only slightly higher than the ambient temperature (approximately 20–22 °C), and heat is dissipated along the upper jig and the surrounding sample holder, resulting in a relatively uniform and low temperature distribution. In contrast, after the friction coefficient rises sharply (Figure 10b), a localized hot spot appears at the contact interface, with the maximum temperature reaching approximately 29 °C, indicating that a large amount of frictional heat is generated in a very short time and cannot be dissipated efficiently through thermal conduction.

Figure 10c further presents the continuous change in temperature with sliding time for the three types of samples. All samples exhibit a similar trend: the temperature remains nearly constant at the ambient level (~18–19 °C) for most of the test, and then increases rapidly once the friction coefficient rises sharply. However, the onset time of the temperature rise differs significantly among the samples. The C-only sample shows the earliest temperature surge (at approximately 22,000 s), followed by the C-LST sample (at approximately 30,000 s), while the LST-C sample exhibits the latest temperature rise (at approximately 35,000 s). This indicates that the LST-C sample maintains a stable low-friction state for the longest duration, thereby delaying the generation of excessive frictional heat. The sudden temperature increase corresponds well with the sharp rise in friction coefficient and the breakdown of the DLC coating, confirming that the failure of the coating leads to direct asperity contact between the ball and the substrate, resulting in severe frictional heating.

### 3.3. Microscopic Morphology of the Wear Debris

Figure 11 shows the microscopic morphology and elemental analysis of the worn surfaces of different samples under dry friction and starved lubrication with a sliding speed of 0.31 m/s. The width of the wear scar under starved lubrication is narrower than that under dry friction. Under starved lubrication, the overall Fe signals are somewhat lower and C/W signals higher for the same sample type, indicating that the PAO4 oil provides some additional protection and helps prolong coating life, though the relative ranking among samples remains unchanged. Figure 11a,a’ (Areas A and D) show that C-only samples has the strongest Fe and O signals and the weakest C and W signals, indicating severe DLC coating removal, extensive substrate exposure, and significant oxidation. This correlates with their highest wear rates and shortest stable friction periods. Figure 11b,b’(Areas B and E) show intermediate behavior of C-LST samples that Fe and O signals are reduced compared to C-only, while C and W signals are more pronounced, suggesting partial coating retention. LST-C samples (Areas C and F in Figure 11c,c’) exhibit the strongest C and W signals and the weakest Fe and O signals, confirming the best coating retention, minimal substrate exposure, and least oxidation. This explains their longest stable friction period and lowest wear rate among the three sample types.

Figure 12 shows the micro morphology and EDS analysis of the wear debris of the samples. It can be seen that the wear debris from the LST-C and C-LST samples has smaller particle sizes than that of the C-only samples. The elemental analysis of the wear debris shows that the C-LST sample has the lowest content of C element (16.7 wt.%) and the highest content of Fe element (66.9 wt.%), while the LST-C sample has the lowest Fe element content (53.4 wt.%) and the highest contents of C and O elements (23.1 wt.% and 19.8 wt.%, respectively). The C-only sample exhibits intermediate values, with a C content of 18.6 wt.%, an Fe content of 60.6 wt.%, and an O content of 16.7 wt.%. These quantitative results indicate that more 45-steel substrate may get involved in the friction process of the C-LST sample than that of the LST-C sample.

## 4. Discussion

The friction coefficient initially maintains a low-stable state, then gradually increasing, and finally suddenly rising sharply during the tribological test both under dry friction and starved lubrication. In the initial stage of wear, the existence of DLC coating has an effect of solid lubrication, so the friction coefficient is low and stable, as shown in Figure 6 and Figure 8. As the sliding time increases, the DLC coating is deformed and the continuity of the coating is disrupted by abrasive particles. Meanwhile, dimpled textures could trap the abrasive debris (Figure 7), reducing the further abrasive wear, thereby the C-LST and LST-C samples have lower friction coefficients and wear rates than that of C-only samples. In starved lubrication, the dimpled textures could also store lubrication oil, meaning in the friction process the oil can be squeezed out, providing secondary lubrication [28,29]. Therefore, the C-LST and LST-C samples have a longer stable state than that of C-only samples. Finally, when the dimples are filled with debris and the coating is broken down, there is no effective lubrication in the contact area, leading to the sharp increase in the friction coefficient. A lot of heat is generated in a short time, which is difficult to disperse though thermal conduction. As a result, the temperature around the contract area rapidly increases as shown in Figure 11. It is noteworthy that the wear rate of the C-only sample decreases with increasing sliding speed under both dry friction and starved lubrication. At higher sliding speeds, the increased frictional heating promotes the graphitization of the DLC coating, transforming sp^3^-hybridized carbon bonds into sp^2^-hybridized graphitic structures that act as a solid lubricant [30,31]. Thermal softening at higher sliding speeds may redistribute the contact stress over a larger area, reducing peak stress concentrations and delaying coating failure. These mechanisms collectively contribute to the observed decrease in wear rate of the C-only sample with increasing sliding speed.

Compared to C-LST sample, LST-C sample has a longer stable state and lower wear rate. On the one hand, the LST may disrupts the continuity of DLC coating, resulting in spalling of coating more easily from around the texture [32,33]. On the other hand, LST could have a positive effect on the deposition of DLC coating, which has a better binding with the substrate [34,35]. It may also be evident from Figure 12 that C-LST sample debris has the highest content of Fe element, which indicates that spalling of coating leads to much more 45-steel substrate involved in the friction process. 

The results of the present study are consistent with previous reports on the combination of LST and DLC coatings. Zhang et al. [29] achieved an 89% reduction in wear rate using a texture/DLC system, while Bai et al. [30] reported that a textured surface with DLC coating reduced the stable friction coefficient by 59% compared to a smooth surface under lubricated conditions. Shi et al. [28] and Peng et al. [32] demonstrated that optimized texture geometry can further enhance the tribological performance by dispersing contact stresses and inhibiting crack propagation. However, none of these studies systematically investigated the influence of processing sequence. The present study directly compared LST-C and C-LST configurations to investigated the influence of processing sequence. The results show that the LST-C sequence is superior, with a wear rate reduction of 33.5%–96.4% compared to the C-only sample and a stable friction duration 1.25–6 times longer. This finding provides new insights into the design of duplex-treated surfaces and highlights the importance of processing sequence in achieving optimal tribological performance.

In summary, the superior tribological performance of the LST-C sample can be attributed to a synergistic combination of mechanical interlocking, debris entrapment, and lubricant storage. The dimples act as reservoirs for wear debris, capturing particles that would otherwise cause abrasive wear and fragmenting them into smaller sizes. Under starved lubrication, the dimples also store and gradually release lubricating oil, providing secondary lubrication and extending the stable low-friction period. 

## 5. Conclusions

(1) The change in friction coefficient follows the same trend both under dry friction and starved lubrication, that is initially maintaining a low-stable state, then gradually increasing, and finally suddenly rising sharply.

(2) Compared to the coating-only sample, the duplex-treatment (C-LST and LST-C) can further reduce the friction coefficient and wear rate at different sliding speeds. The LST-C samples have the best performance both under dry friction and starved lubrication, which increases the duration time of the stable-stage by 1.25~6 times for the C-only samples, and also reduces the wear rate by 33.5~96.4%.

(3) The processing sequence of the DLC coating and LST has an impact on the tribological properties of the samples. The LST-C samples have better performance in friction-reduction and wear resistance than the C-LST samples.

## Figures and Tables

**Figure 1:**
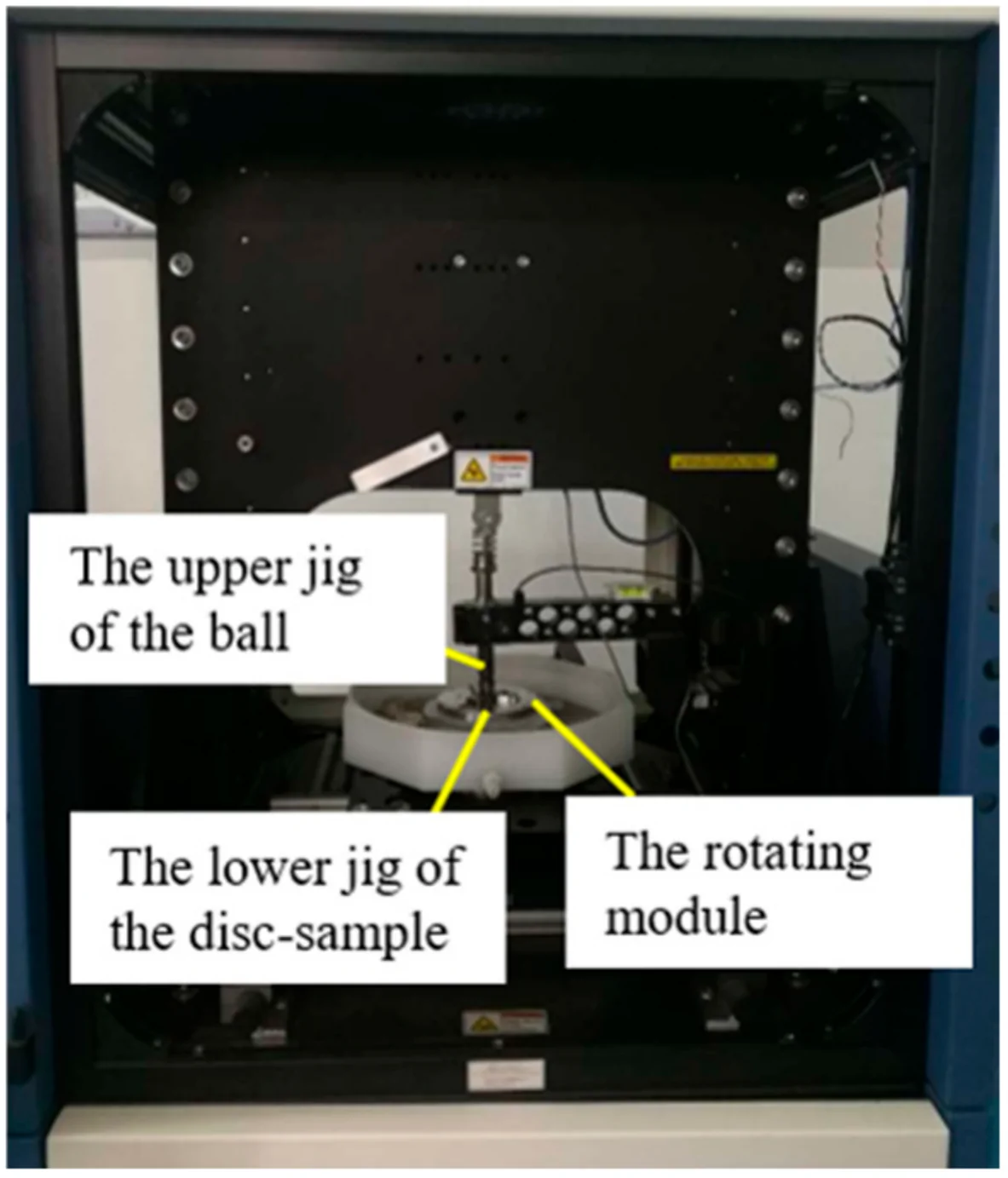
The test setup of the MFT-5000 tribometer.

**Figure 2:**
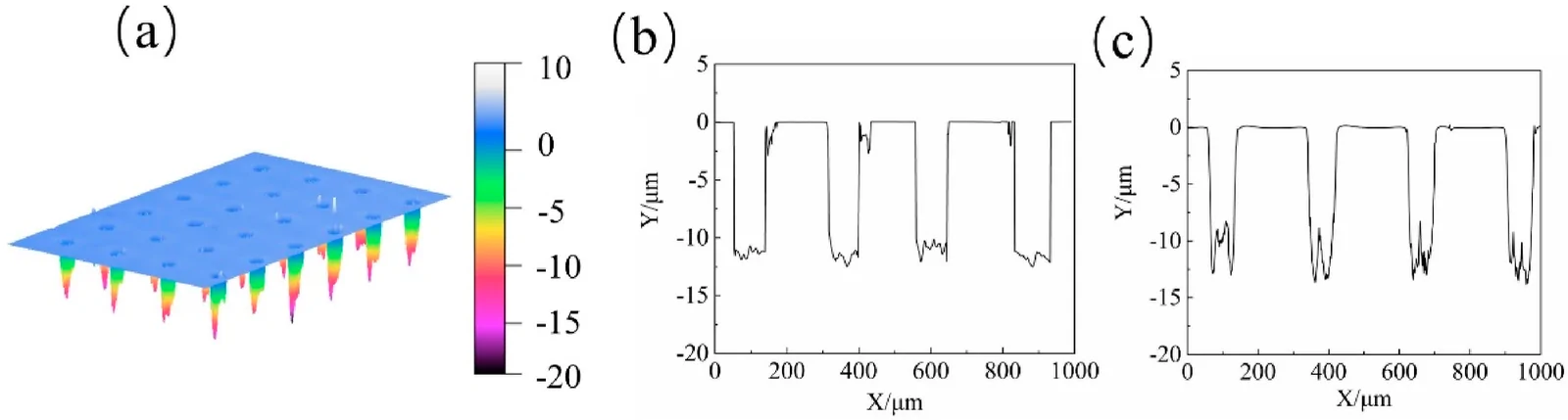
Surface topography and profiles of the textured samples: (**a**) 3D topography map of the LST-C sample; (**b**) cross-sectional profile of the LST-C sample; (**c**) cross-sectional profile of the C-LST sample.

**Figure 3:**
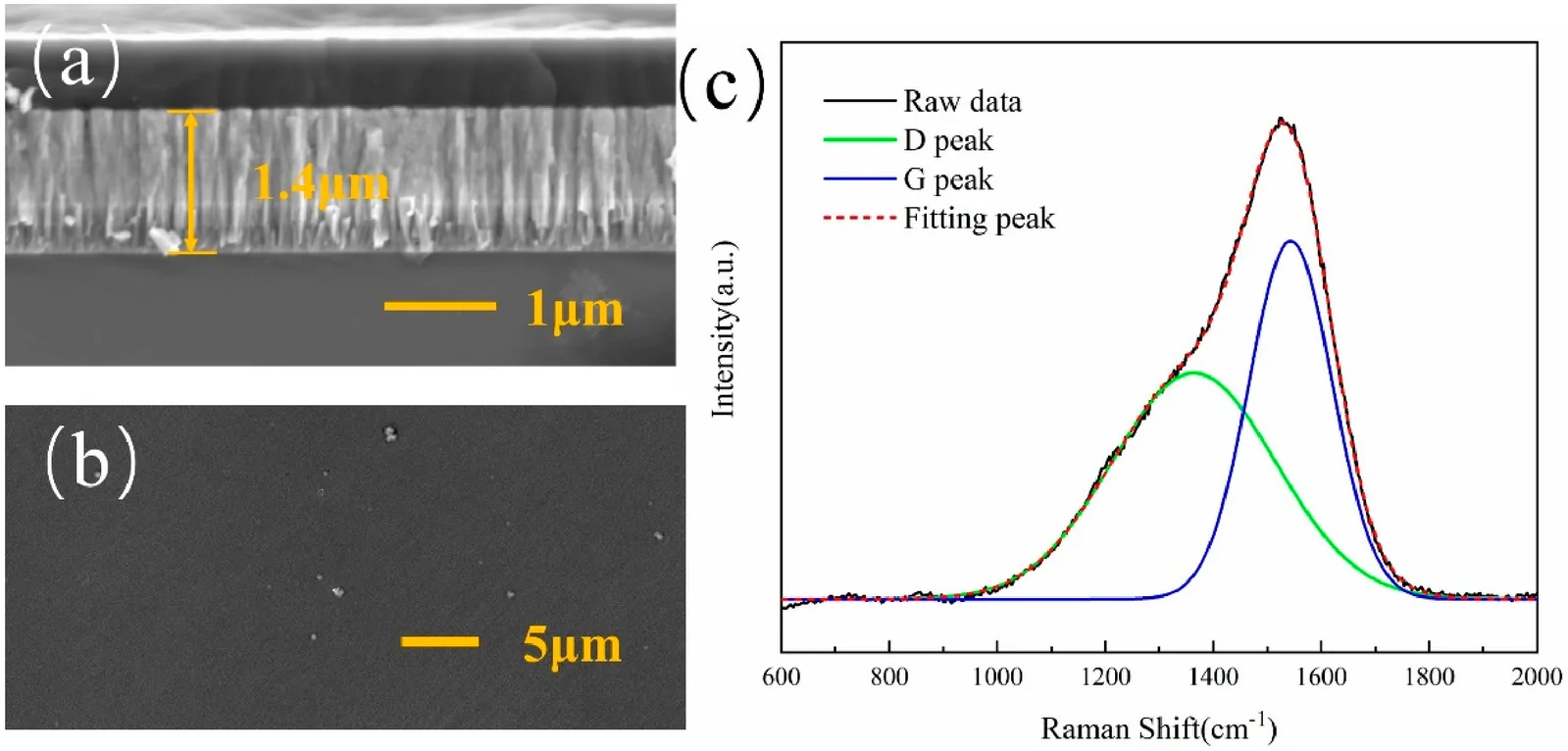
Characterization of DLC coating (C-only sample): (**a**) cross-sectional SEM image; (**b**) SEM morphology of the coating surface; (**c**) Raman image.

**Figure 4:**
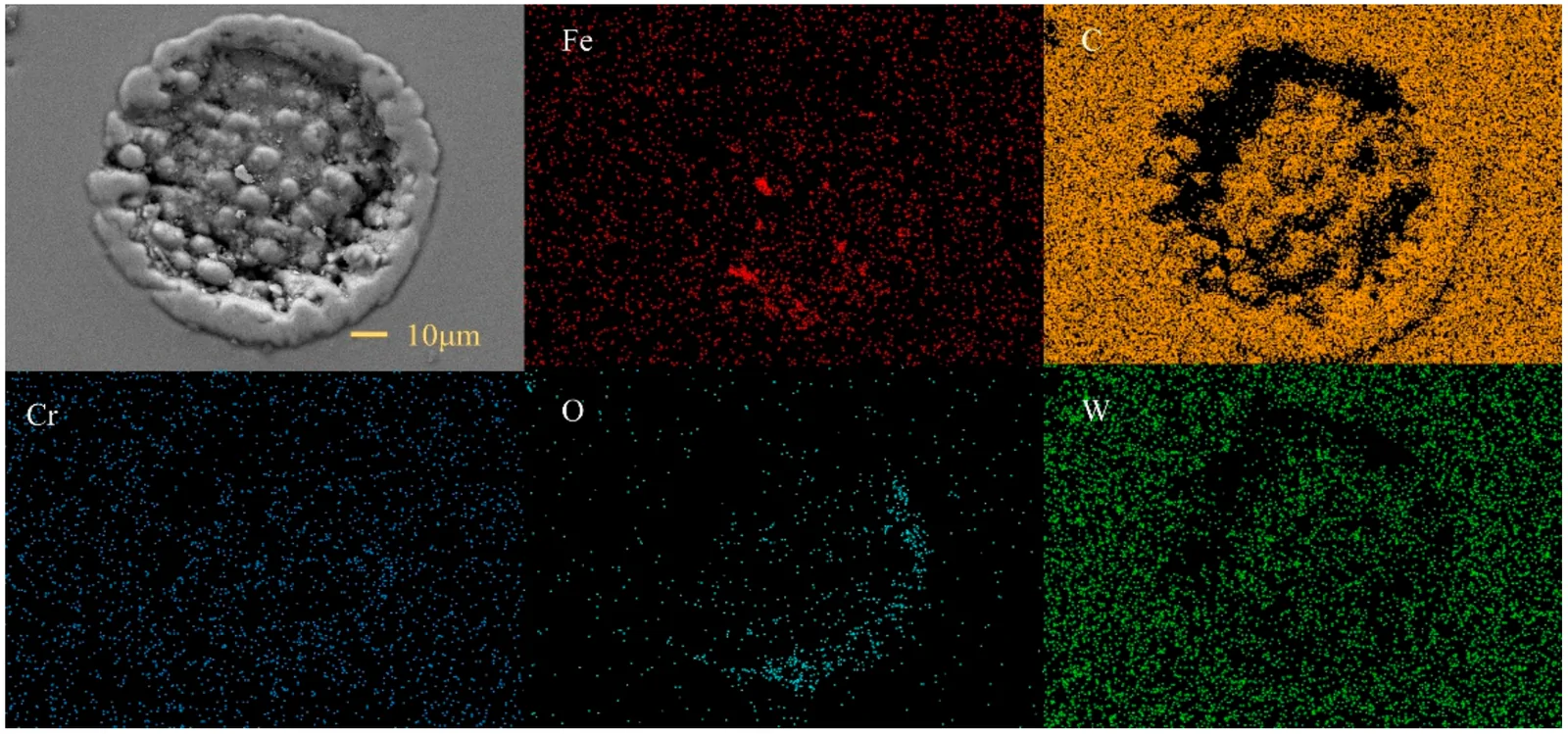
EDS image of C-LST sample.

**Figure 5:**
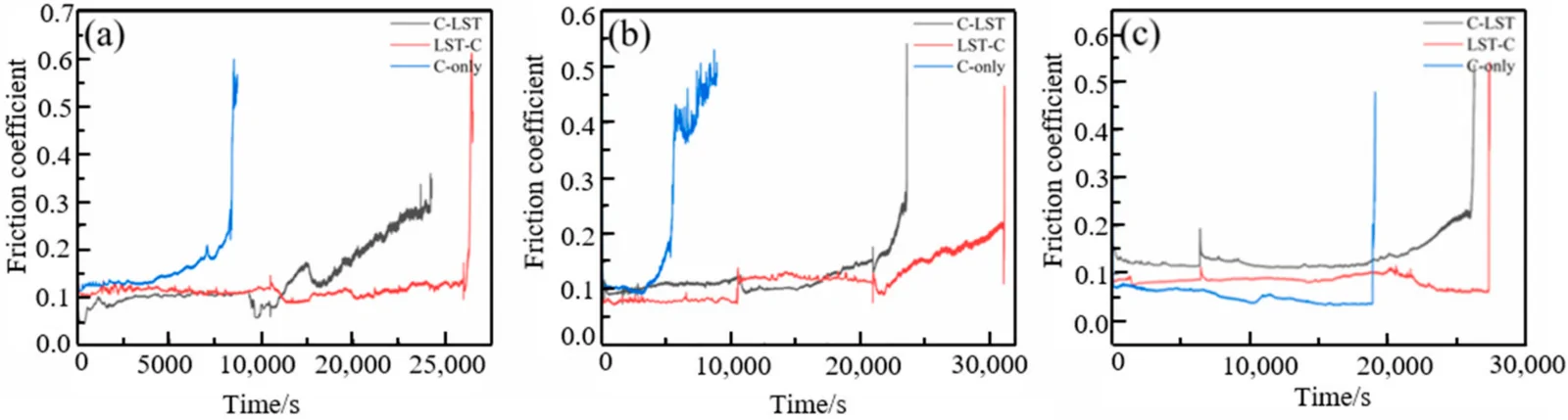
Friction coefficients of the samples in different sliding speeds under dry friction: (**a**) 0.21 m/s; (**b**) 0.31 m/s; (**c**) 0.42 m/s.

**Figure 6:**
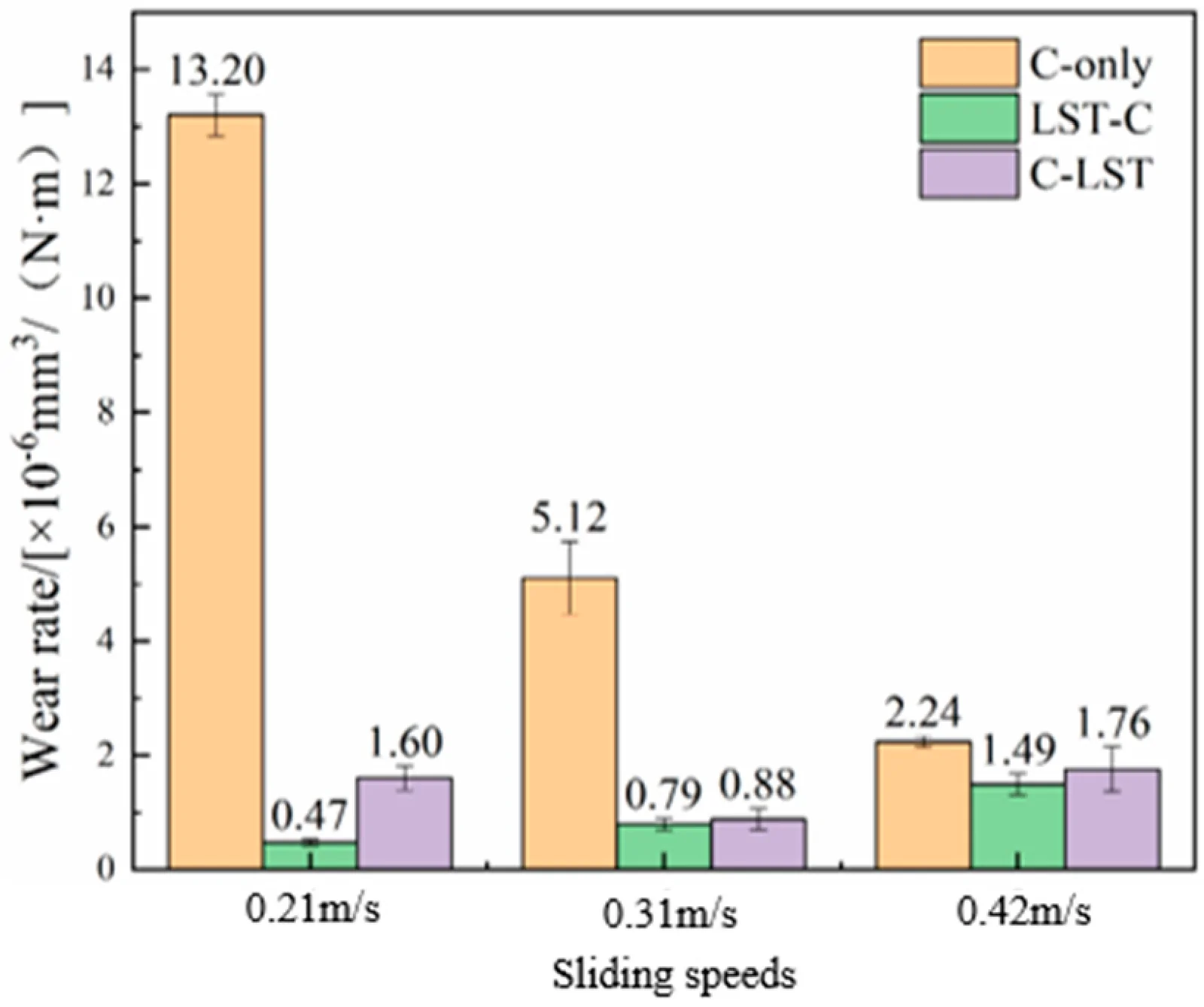
Wear rate of the samples in different sliding speeds under dry friction.

**Figure 7:**
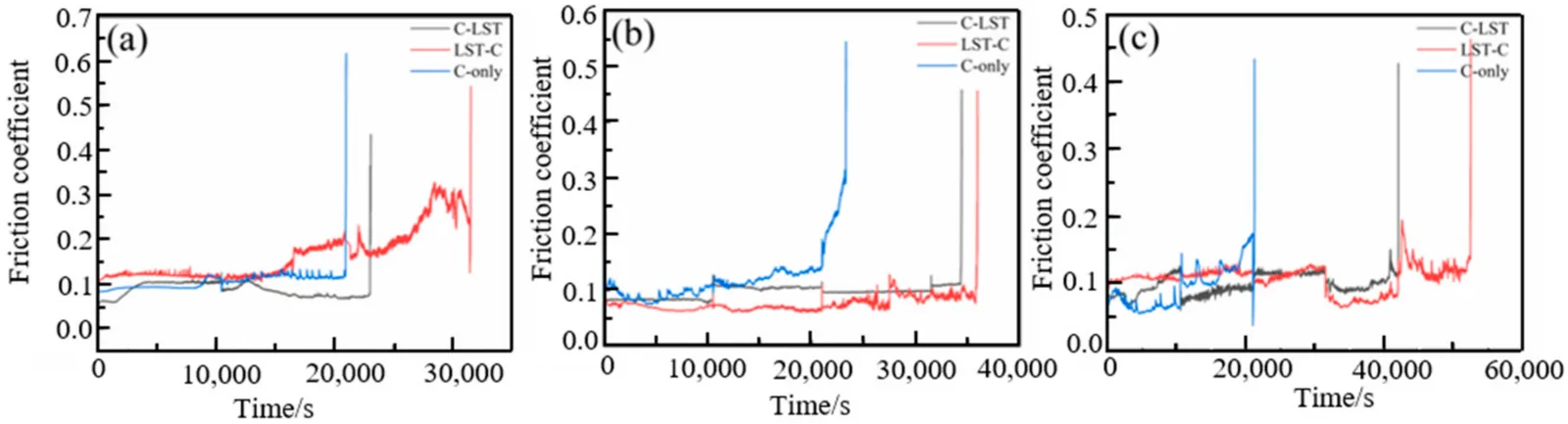
Friction coefficients of the samples with different sliding speeds under starved lubrication: (**a**) 0.21 m/s; (**b**) 0.31 m/s; (**c**) 0.42 m/s.

**Figure 8:**
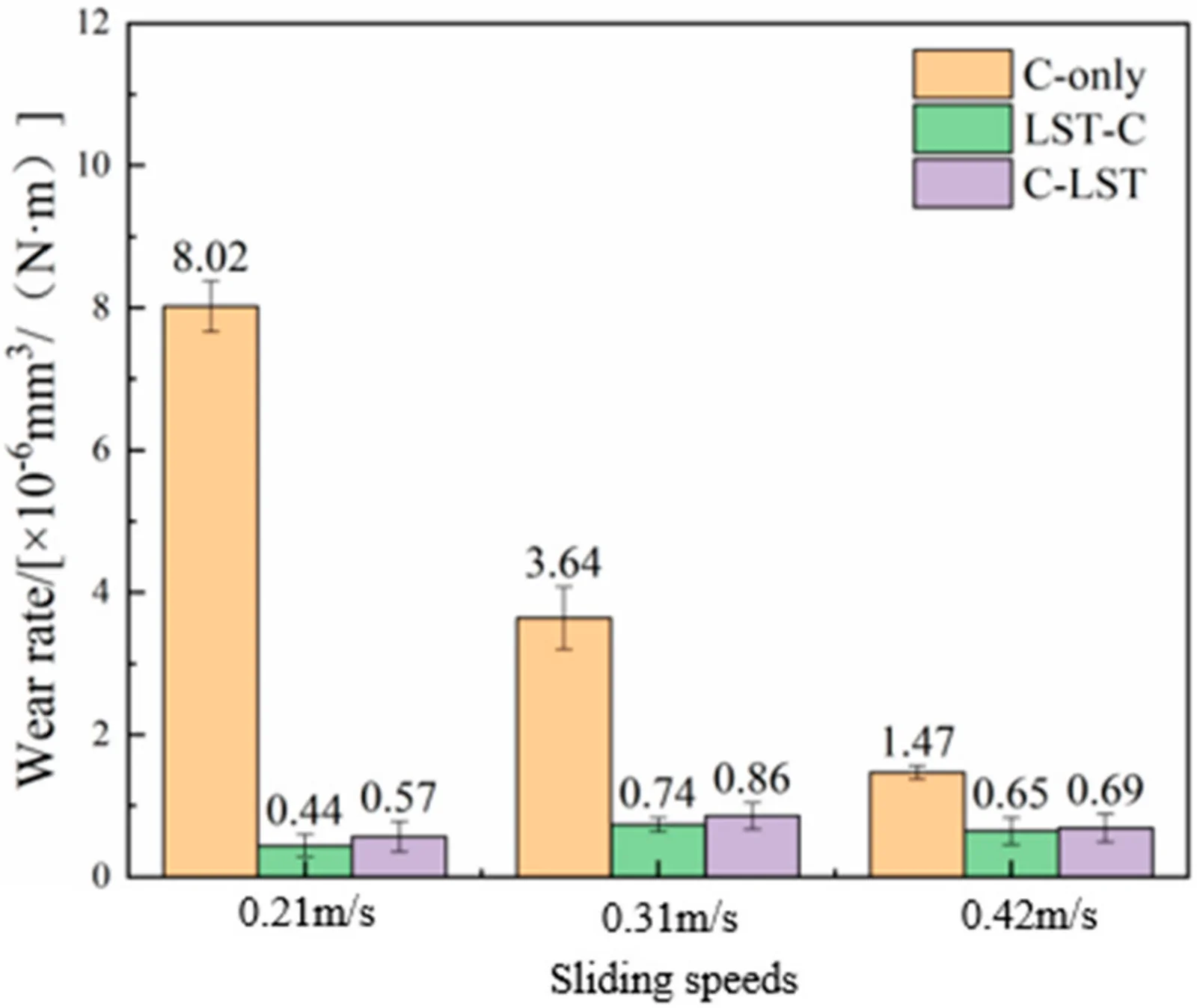
Wear rate of the samples for different sliding speeds under starved lubrication.

**Figure 9:**
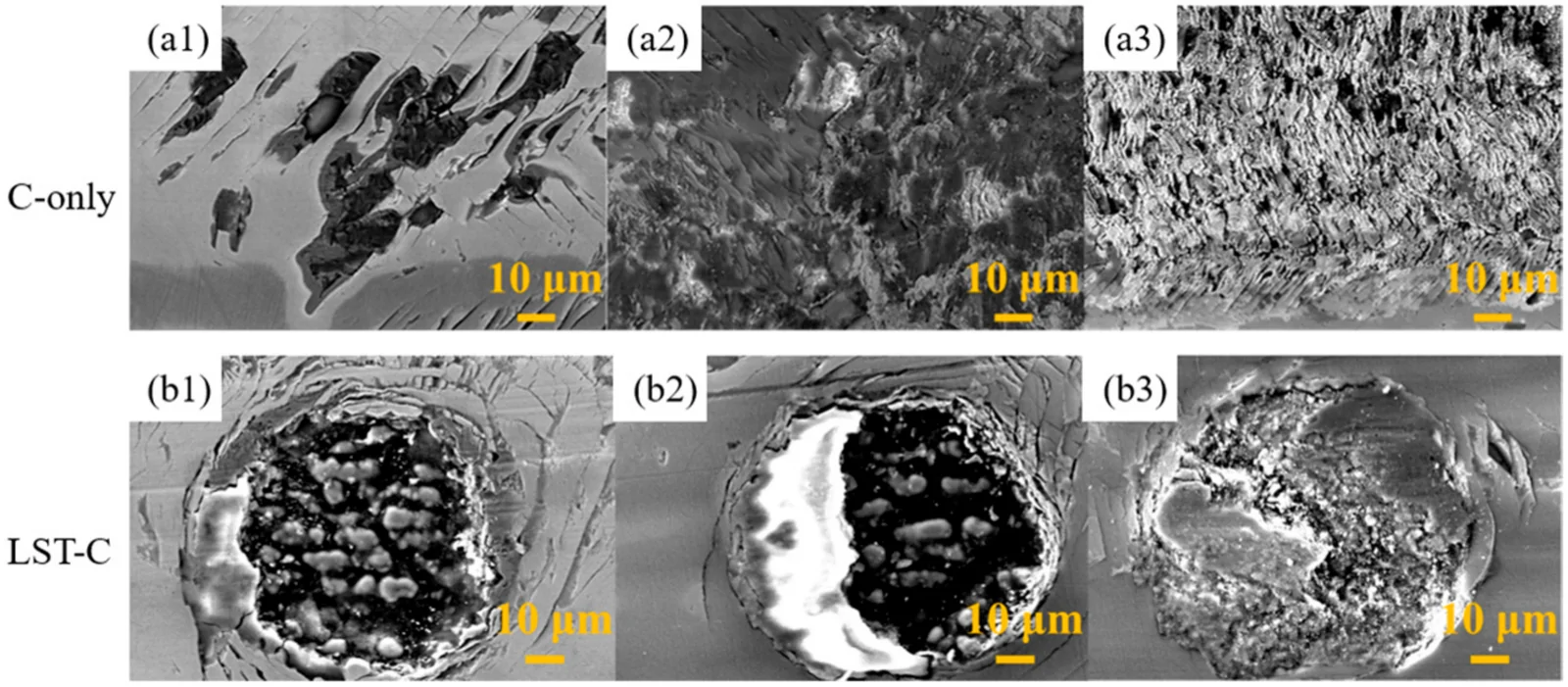
Microscopic morphologies of C-only and LST-C samples at different sliding times under starved lubrication: (**a1**) 5,000 s; (**a2**) 15,000 s; (**a3**) 21,000 s; (**b1**) 15,000 s; (**b2**) 21,000 s; (**b3**) 30,000 s.

**Figure 10:**
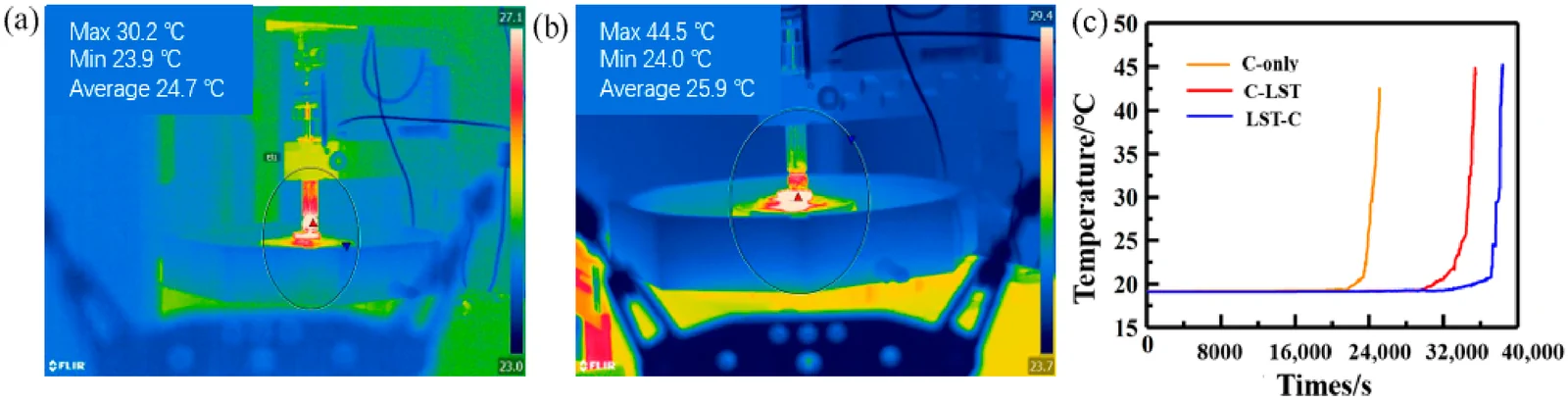
Temperature of the sample surfaces: (**a**) before the friction coefficient rising sharply; (**b**) after the friction coefficient rising sharply; (**c**) the change in the temperature in the friction process.

**Figure 11:**
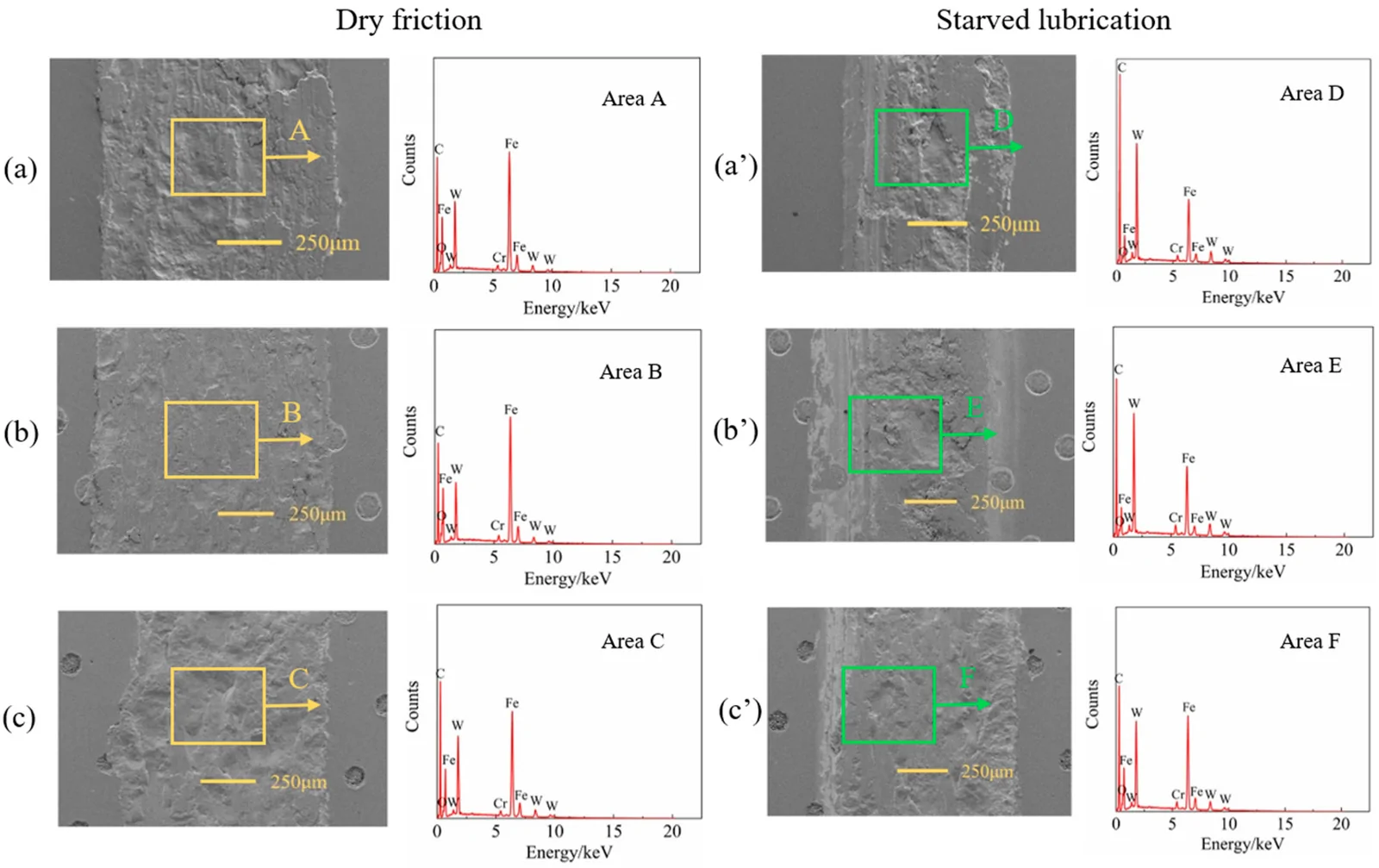
Microscopic morphology and elemental analysis of the worn surfaces of different samples under dry friction and starved lubrication at a sliding speed of 0.31 m/s: (**a**) and (**a’**) C-only; (**b**) and (**b**’) C-LST; (**c**) and (**c’**) LST-C.

**Figure 12:**
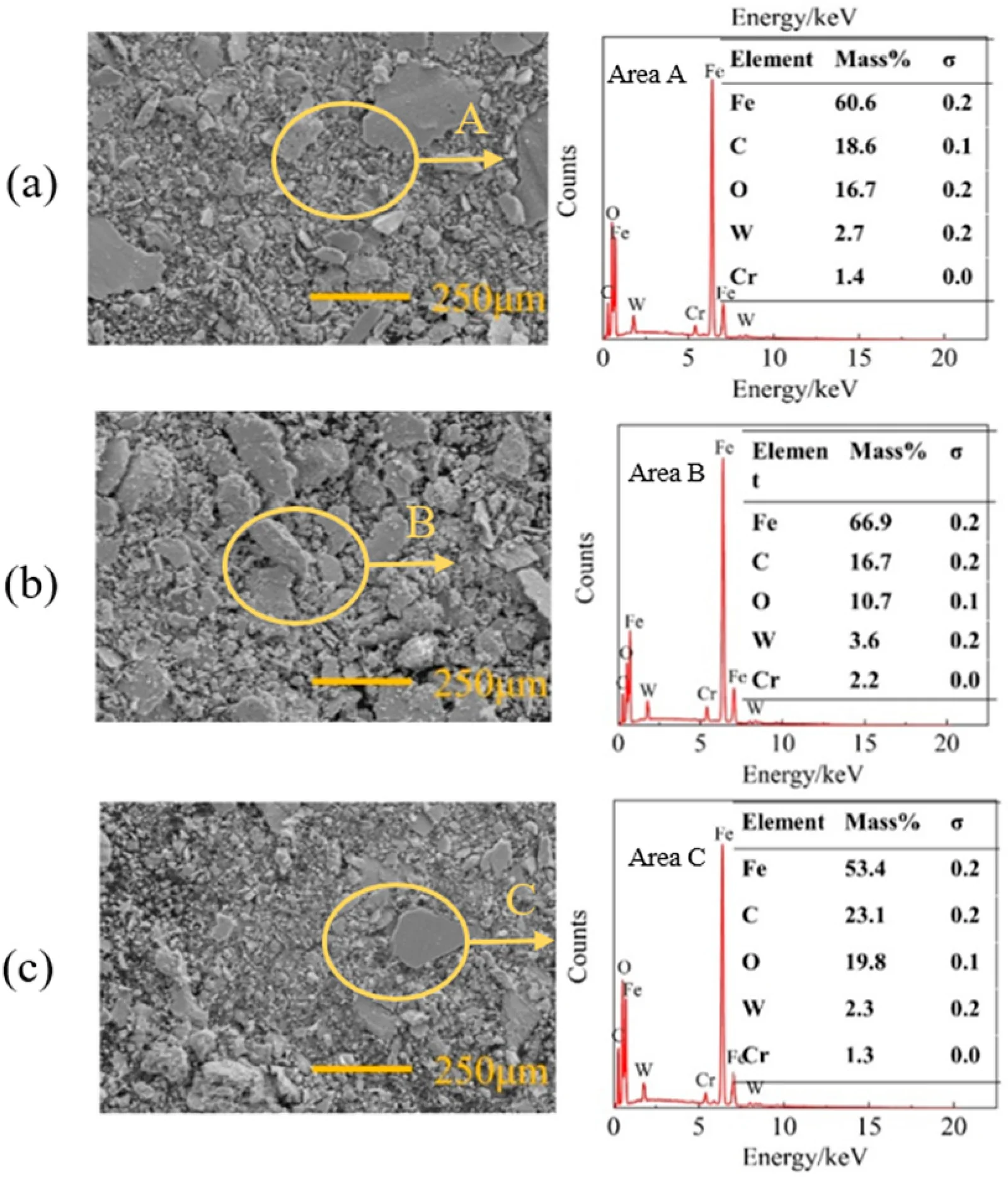
Microscopic morphology of grinding debris and corresponding EDS analysis of the samples: (**a**) C-only; (**b**) C-LST; (**c**) LST-C.

**Table 1:** Chemical composition of 45-steel (wt%).

C	Si	Mn	P	S	Cr	Ni	Cu
0.45	0.22	0.56	0.013	0.003	0.043	0.009	0.035

**Table 2:** Laser processing parameters.

Laser Frequency kHz	Laser Diameter μm	Wavelength nm	Pulse Width ns	Output Power W
50	12–16	355	20	0.177

**Table 3:** Summary of specimen variants and processing sequences.

Specimen Designation	Processing Sequence	Description
C-only	DLC coating only	45-steel substrate → polishing → ultrasonic cleaning → Ar^+^ ion cleaning → W interlayer deposition → DLC coating deposition. No laser surface texturing was performed.
LST-C	LST followed by DLC coating	45-steel substrate → polishing → ultrasonic cleaning → laser surface texturing (square array of micro-dimples, 10% density, ~100 μm diameter, ~10 μm depth) → gentle polishing to remove rim bulges → ultrasonic cleaning → Ar^+^ ion cleaning → W interlayer deposition → DLC coating deposition.
C-LST	DLC coating followed by LST	45-steel substrate → polishing → ultrasonic cleaning → Ar^+^ ion cleaning → W interlayer deposition → DLC coating deposition → laser surface texturing (same texture parameters as LST-C) → gentle polishing to remove rim bulges → ultrasonic cleaning.

**Table 4:** The physical properties of PAO4 oil.

Oil	Specific Gravity	Pour Point (°C)	Flash Point (°C)	Kinematic Viscosity (cst)	
				40 (°C)	100 (°C)
PAO4	0.820	−70	215	16.8	3.9

## Data Availability

The original contributions presented in this study are included in the article. Further inquiries can be directed to the corresponding author.
